# Creating a Perioperative Glycemic Control Program

**DOI:** 10.1155/2011/465974

**Published:** 2011-09-06

**Authors:** Sara M. Alexanian, Marie E. McDonnell, Shamsuddin Akhtar

**Affiliations:** ^1^Department of Endocrinology, Diabetes and Nutrition, Boston University Medical Center, 88 East Newton Street, Evans 201, Boston, MA 02118, USA; ^2^Department of Anesthesiology, Yale University School of Medicine, New Haven, CT 06510, USA

## Abstract

Hyperglycemia in the surgical population is a recognized risk factor for postoperative complications; however, there is little literature to date regarding the management of hyperglycemia in the perioperative period. Here, we detail the strategies that our institutions have employed to identify and treat hyperglycemia in patients with diabetes who present for surgery. Our approach focuses on the recognition of hyperglycemia and metabolic abnormalities, control of glucose levels via insulin infusion when needed, monitoring for hypoglycemia and a comprehensive multidisciplinary approach that provides standardized recommendations for patients at all points in care as they transition from the preoperative clinic into the operating room, and then into the hospital.

## 1. Background

Studies have demonstrated that hyperglycemia occurs in a significant percentage of hospitalized patients; seventy percent of patients with diabetes admitted with acute coronary syndrome and 80% of cardiac surgery patients in the perioperative period may develop hyperglycemia [1]. Over the past few decades, there has been mounting evidence that hyperglycemia in hospitalized patients leads directly to adverse consequences. In particular, the literature indicates a role for glycemic control in surgical patients, where postoperative hyperglycemia is associated with an increased risk of infection, renal and pulmonary complications, and also mortality [2–7]. More recent studies have addressed the effects of hyperglycemia perioperatively and confirmed similar associations [8–11]. One study demonstrated that for every 20 mg/dL increase in the mean intraoperative glucose, the risk of an adverse outcome increased by more than 30% [8]. 

While the majority of intervention trials for glycemic control have taken place in critical care settings and in the cardiac surgery population (and have been extrapolated to other clinical situations), some data is now emerging for general hospital wards and in patients undergoing noncardiac surgery [12, 13]. However, there are few clinical trials that have specifically studied the intraoperative period. In one intervention trial in patients undergoing cardiac surgery, attempting to achieve intraoperative tight glycemic control (<100 mg/dL) did not show improvement in outcomes when compared to good glycemic control (<200 mg/dL) [14]. However, in another study by Subramanian et al. [12], involving 236 patients undergoing vascular surgery, subjects randomized preoperatively to a continuous intravenous insulin infusion protocol (goal glucose range 100–150 mg/dL) versus an intermittent intravenous bolus protocol (treatment if >150 mg/dL) had a lower rate of perioperative myocardial infarction. This trial, though, was underpowered. While strong evidence to support specific insulin strategies in the operating room are lacking, practitioners have recognized that intraoperative glycemic control assists in achieving early postoperative glycemic control. Glucose control on the hospital floor can take several days to accomplish even when patient-tailored subcutaneous insulin programs are utilized [13, 15, 16], as there are many factors that can effect glycemic control in hospitalized [17] and critically ill patients [18].

Based on available data, both of our institutions have independently developed protocols to achieve rapid and safe glycemic control via intravenous insulin in patients presenting for surgery. This therapy serves as a bridge to subcutaneous insulin to be continued during the hospital stay. Here, we describe our experience creating and implementing these protocols. The discussion below relates to glucose control in patients with diabetes to be admitted to the hospital after surgery. The topic of ambulatory surgery will not be addressed. 

## 2. Laying the Groundwork and Designing a Protocol

Because the perioperative time period involves care by multiple and different physician groups, a multidisciplinary team approach is key to the creation of a successful protocol. Depending on institutional practice, this may include representatives from endocrinology, anesthesiology, surgery, nursing, preoperative clinic, pharmacy, and information technology (IT). Every transition point for the patient should be addressed, from the preoperative assessment clinic with standardized recommendations for antihyperglycemic medications before surgery, to transition to in-hospital postoperative care as well as discharge preparation and education. It is important that a standardized protocol for the treatment of hypoglycemia be included whenever a hyperglycemia protocol is instituted.

 Preoperative control of glucose makes intuitive sense. However, the ideal range and duration of glucose control prior to elective surgery has not been determined. No prospective study has been conducted to date to demonstrate that preoperative glucose control improves perioperative outcomes. A recent retrospective study involving more than 55,000 patients failed to demonstrate an association between preoperative HbA1c and postoperative infection rate [11]. Furthermore, recent trials in *nonoperative* clinical situations have failed to conclusively demonstrate that tight glucose control as measured by HbA1c leads to significantly better cardiovascular outcomes [19]. It is because of this data that the main focus has remained on achieving glucose control in the hospital and upon discharge. 

Due to its quick action and short half life, intravenous (IV) insulin is the preferred choice for rapid correction of hyperglycemia. While providers may be more familiar with ordering subcutaneous regular insulin or rapid-acting insulin analogs, it will take several hours for these insulins to have their peak effect and to be metabolized, which both limits the frequency at which the medication can be redosed and increases the amount of time the patient may require monitoring (e.g., the duration of action of subcutaneous regular insulin is 6–10 hours). Furthermore, many factors can affect insulin absorption in the perioperative period and in critically ill patients [7]. The result is an increased potential for overlapping dose effects, administration timing errors, and unexpected hypoglycemia. Thus, it is now recommended that insulin be administered intravenously in the perioperative period and for critically ill patients. Many institutions with critical care units already have protocols for glycemic control using intravenous insulin infusions, and these can often be adapted for use in the operating room. If this is not available, evidence-based insulin infusion protocols have been published [20–27]. Protocols that incorporate the current glucose value, the previous glucose values, and the rate of the infusion are preferred [28, 29]. However, because recommended goal glucose ranges have changed over time, careful attention must be paid to the protocol target range. Recent trials and meta-analyses have failed to show a benefit in attempting to achieve normoglycemia (e.g., 80–110 mg/dL) in diverse groups of critically ill patients and in the operating room [14, 30–33], and, based on available data and concerns about hypoglycemia, a more moderate goal now seems prudent. The American Association of Clinical Endocrinologists and the American Diabetes Association recommend a glucose target of 140–180 mg/dL in critically ill patients [34]. Many other organizations have made similar recommendations (Table 1) [35–39]. 

It is important that a plan be in place to treat hyperglycemia after the patient leaves the operating room (OR). There is often confusion on the part of the providers who assume that if a patient is started on an insulin infusion that they must be admitted to the hospital or even the intensive care unit in order to continue the infusion after surgery. Instead the infusion should be viewed as a temporary intervention to rapidly achieve metabolic control. Patients who would normally be transferred to the floor after a procedure should be assessed for transition to an appropriate hospital regimen, such as basal-bolus insulin [34]. There are several decision points in the perioperative time (arrival in the preoperative area, transfer to the OR, and transfering out the postanesthesia care unit) when the patients' therapy will need to be adjusted. Working to streamline and standardize these decision points will limit delays and an unnecessary increase in hospital resources and staff time. 

Other practical issues of the protocol will need to be addressed including the availability of glucometers and premade IV insulin bags. IT support can assist in creating standardized order sets for those institutions with computerized orders.

## 3. Hypoglycemia

Hypoglycemia and the fear of hypoglycemia remain a major barrier in the care of hospitalized patients. In prospective studies, the incidence of significant hypoglycemia is reported to be up to six times higher in intensive glucose control groups. Recent data has demonstrated a relationship between hypoglycemia, morbidity, and mortality [40–44], though whether the hypoglycemia is causal, or a sign of critical illness has yet to be established [43, 45]. Physicians have a heightened and appropriate concern for patients who are sedated as they will be unable to report symptoms, and the signs of hypoglycemia may be masked. For patient safety, a standard treatment algorithm for hypoglycemia must be included as part of the glycemic protocol. Patients on intravenous insulin infusions in the OR should have a glucose checked at a minimum every sixty minutes, and more often as clinically indicated. Monitoring methods can include the use of point-of-care glucose meters or blood samples such as venous blood gases during the procedure. One of the drawbacks of glucose meter use is the variance between meter readings and laboratory sample (allowed to be up to 20% by FDA regulations). The FDA is currently reviewing these limits, and revised regulations may be forthcoming. Many patient factors are known to affect the accuracy of the reading, including pH, oxygenation status, and anemia among others, and this has been shown to be a particular issue in critically ill patients [46, 47]. Additionally, when a glucose value is in the hypoglycemic range, the accuracy is further decreased [47–49]. Caregivers using these devices need to be educated about their limitations and a value that is not consistent with the clinical picture needs to be verified by a central laboratory method. This issue is another reason not to target a glucose value in the normoglycemic range but only treat if glucose is more than 180 mg/dL [39].

## 4. Surgical Cancellations

Currently no evidence-based guidelines exist regarding when to cancel a surgical procedure due to hyperglycemia. Given the multitude of patient factors involved as well as the variety of surgical procedures and procedure urgency, it is unlikely that recommendations based on outcomes will be forthcoming. Providers need to weigh several issues when considering this question. First of all, the urgency of surgery should be considered. Secondly, hyperglycemia could represent an unstable metabolic state, such as diabetic ketoacidosis, which should be rapidly assessed in the preoperative area. Elective surgery in unstable metabolic state is not recommended. Furthermore, the chronic glycemic state of the patient should be considered. In our experience, most patients who present for elective surgery with a glucose >300 mg/dL have had similar values documented as an outpatient and are a representation of chronically poor control, as opposed to a new illness. In this situation, there are opportunities for providers to identify and address the problem prior to the patient arriving in the preoperative area. Another consideration is that the hyperglycemia may be caused by the illness for which the patient presented for surgery (for example, osteomyelitis), which would not be expected to improve until the patient undergoes surgery. Providers need, therefore, to assess the patient for stability, the need for the procedure, the risks of the procedure, and the ability of the patient to achieve glucose control if the surgery is postponed. We have used a cutoff of 300 mg/dL (Boston Medical Center) as a trigger in the preoperative area for evaluation for ketoacidosis either via urine ketone dipstick or whole blood chemistry. At Yale New-Haven Hospital, no cutoff value to trigger evaluation for ketoacidosis has been set. It has been left to the discretion of the physician. However, it is recommended to postpone nonurgent/emergent surgery if the glucose is >400 mg/dL. At Boston Medical Center, it is recommend to postpone nonurgent procedures if the glucose is >500 mg/dL, or at the discretion of the physician at lower levels based on the risks and urgency of the procedure.

## 5. Training and Educational Strategies

The multidisciplinary nature of a perioperative protocol necessitates education over the course of time and in different formats. At Boston Medical Center, this included surgical and anesthesiology grand rounds to review data and recommendations and later a joint conference regarding the practical implementation of the protocol. There were nursing in-services as well as training in the use of glucose meters and point-of-care testing with ketostix. Nurses without prior ICU experience also needed training in insulin infusion administration. At all steps, the physician groups were given updates at conferences and via emails. We also developed an educational video that was available for viewing on the hospital intranet. The endocrinology team was trained in the protocol to provide support when issues arose. In order to assess the safety of our protocol and to identify unforeseen issues, a three-month pilot of the protocol was performed in one OR area prior to it being used hospital wide. The leadership group focused on the efficacy at achieving glycemic control and the incidence of hypoglycemia, as well as any needed adjustments to nursing orders before deciding to expand the program. Pilot results have been previously published [50]. At Yale New Haven hospital, similar education was employed, and an initial protocol was tried in the cardiothoracic ICU and then introduced to the perioperative services. We are in the process of analyzing the data for our in-hospital population.  Table 2 summarizes the main challenges that arose during the creation and implementation of these protocols and how they were addressed. 

## 6. Program Descriptions

There is currently a lack of evidence to guide providers regarding the details of perioperative glycemic management. We provide this information to report our experience and inform the literature, not as a formula we wish to recommend as the ideal or only way to approach the issue. Below, we describe the patient flow that occurs at each of our institutions for an example patient. A comparison of the two programs is provided in Table 3.

**Figure 1:**
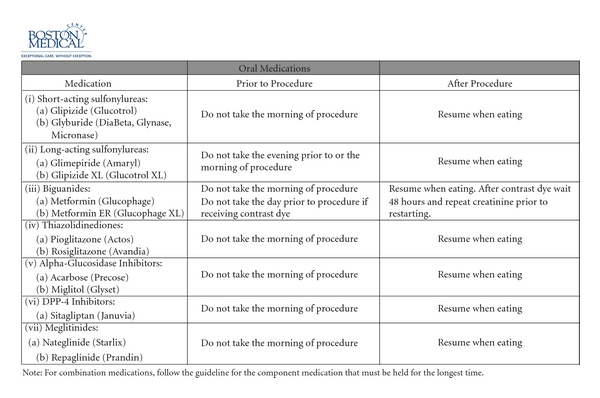
Boston Medical Center Guidelines for Pre-procedure Outpatient Management of Antihyperglycemic Medications for Procedures that Require “NPO” Status.

**Figure 2:**
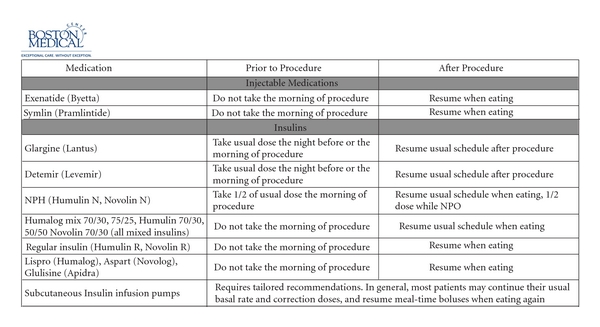
Boston Medical Center Guidelines for Pre-procedure Outpatient Management of Antihyperglycemic Medications Prior to Procedures that Require “NPO” Status.

Boston Medical Center: the patient is seen in the preoperative care clinic the week prior to surgery. The endocrinology and preoperative clinic have created a standard guideline to adjust medications prior to surgery (Figures 1 and 2). All patients undergoing surgery automatically have an order for perioperative glycemic control in our computerized system cuing the nurse to start the protocol. Upon arrival, the nurse in the preoperative area will check a glucose level on all patients with diabetes. Patients with a glucose level of ≤180 mg/dL proceed to surgery. Patients with a glucose level of 181–300 mg/dL are started on an IV insulin infusion by the nurse prior to the proceeding of the operating room. A 5% dextrose solution is also initiated to decrease the risk of hypoglycemia. The goal glucose range on our infusion protocol is 120–180 mg/dL. The anesthesiologist is made aware of the treatment in the preoperative area. The infusion is included as part of the presurgical WHO checklist to assure all staff are aware of the therapy, and the anesthesiologist continues insulin titration according to the protocol in the operating room. On arrival in the postanesthesia care unit, the endocrine fellow is paged. The fellow provides recommendations for a subcutaneous insulin program while in the hospital. Patients with a glucose level >300 mg/dL prior to surgery have a metabolic evaluation for ketones or for acidosis prior to starting the infusion and proceeding to the operating room. As mentioned above, we recommend postponing nonurgent procedures when the glucose is >500 mg/dL or at the discretion of the provider. For patients with type 1 diabetes or patients on an insulin pump, the endocrinology fellow is paged prior to surgery. It should be noted that it took one year for our multidisciplinary team to create the protocol, ensure the necessary equipment, and perform the needed education prior to the three-month trial pilot. After this, adjustments were made to the protocol, the education was expanded, and it took nine months before the protocol was used hospital wide.

**Figure 3:**
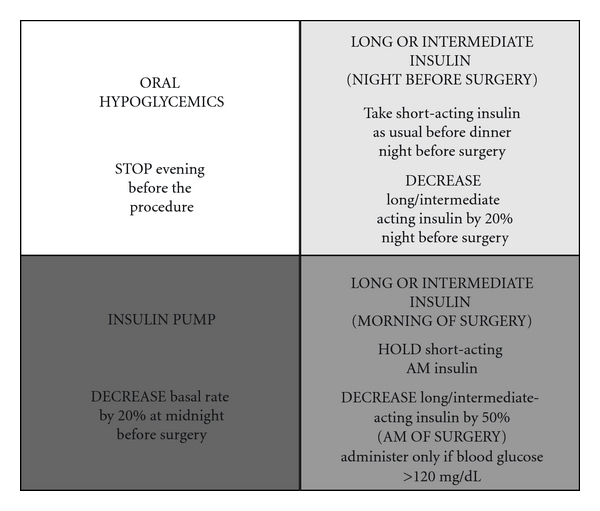
Recommendations for adjusting antiglycemic medications (oral hypoglycemic, long- and short-acting insulin and insulin pump infusion rates) preoperatively from Yale New Haven Hospital (see text for details).

Yale-New Haven Hospital: the protocol was developed by the multidisciplinary perioperative team, which includes anesthesiologists, endocrinologists, intensivists, nurses, and administrators. Patients are seen in the preoperative anesthesia clinic from a day to a few weeks prior to surgery. They are advised to adjust their antihyperglycemic medications based on the following guideline (Figure 3). Typically, the resident physician or the nurse who evaluates the patient gives these instructions. Patients who are on oral antihyperglycemic medications are advised to discontinue their medications the night before surgery. No oral hypoglycemic medications are administered or advised on the morning of surgery. Medications are reinstituted after the patient has resumed a normal diet. For patients who are taking short or long acting insulins adjustment of the insulin should take into account the timing of their insulin regimen. Patients who take both evening and morning doses of insulin should take their usual dose of evening short-acting insulin, but reduce their intermediate/long-acting insulin dose by 20% the night before surgery. However the morning of surgery they should omit their morning short acting insulin and reduce the intermediate/long-acting dose by 50% (and take this only if the fasting glucose is more than 120 mg/dL). If patients are using a premixed insulin they are instructed to reduce their evening dose prior to surgery by 20% and hold insulin completely morning of the procedure. 

Patients with type 1 diabetes need some basal insulin at all times. Short/rapid acting insulin alone will not suffice to control blood glucose. Patients are instructed to take and reduce their evening intermediate/long acting insulin by 20% the night before surgery. Those who use AM intermediate/long acting insulin are instructed to take 50% of their usual dose (as long as fasting blood glucose is equal to or more than 120 mg/dL). For patients on insulin pumps, the dosage should be reduced by 20% at midnight before surgery. All patients with diabetes have their glucose checked prior to elective surgery, and insulin therapy is started based on written protocols. Urgent metabolic derangements are assessed if dictated by clinical situation. In contrast to the protocol at Boston Medical Center, at Yale New-Haven Hospital, IV insulin is not started in the preoperative area. Based on a protocol, subcutaneous aspart insulin is administered if the glucose is >200 mg/dL. If the glucose is >400 mg/dL, the anesthesiologist is informed. Elective surgery is discouraged if an acute rise in glucose levels to >400 mg/dL is noted or at the discretion of the provider. The endocrine fellow is consulted if there are any concerns. Glucose levels are measured intraoperatively if the patient has diabetes or has been administered any insulin preoperatively. Intraoperatively insulin is administered intravenously, and glucose is monitored hourly if an insulin infusion is started (for blood glucose >180 mg/dL) or if the patient is on insulin pump. The goal is to maintain the glucose between 120–180 mg/dL intraoperatively. Once the patient arrives in the postanesthesia care unit, the insulin infusion is either continued or transitioned a to basal-bolus program based on the final disposition of the patient. If the patient is on insulin infusion, potassium is also monitored closely. Hypoglycemia treatment is clearly addressed in the glycemic protocol and if any untoward events or side effects of insulin are noted, the endocrinology fellow is consulted immediately.

## 7. Conclusion and Future Directions

With the increasing prevalence of diabetes, practitioners will continue to face the challenge of managing hyperglycemia in patients during all aspects of the hospital stay. We note that especially during the perioperative time where several physician specialties are involved and many care transitions will occur, it is critical that a multidisciplinary team be utilized in addressing this issue. Variation in institutional resources, staff, and existing hospital practice will mean that there is no “one size fits all” approach. Many unanswered questions remain as well as future research opportunities to determine optimal intraoperative glucose targets, when to postpone surgery, and specific populations who may show greater or less benefit from insulin therapy. Here, we provide our experience and await more data to further refine our practice.

##  Disclosure

The authors have nothing to disclose.

## Figures and Tables

**Table 1:** Current recommendations for glycemic control in critically ill patients.

Organization	Year	Patient population	Treatment threshold (mg/dL)	Target glucose
Surviving sepsis campaign	2008	ICU patients	180	<150

American heart association	2009	Patients with Acutecoronary syndrome	180	<140

European society of cardiology	2009	Patients after major noncardiac surgery	180	140–180

Institute of healthcare improvement	2009	ICU patients	180	<180

American diabetes association	2011	ICU patients	180	140–180

American college of physicians	2011	ICU patients/hospitalized patients	180	140–180

**Table 2:** Challenges to creating and implementing a perioperative glycemic control protocol.

Challenges faced	Solutions employed
Consensus of a need for action by key leaders in representative departments and formation of *Task force *for perioperative glycemic control	MD leaders in endocrinology and anesthesiology provided education and interdepartmental outreach (e.g. conferences, consultation) on risks of hyperglycemia and reviewed hospital-specific patient outcomes as well as national data and guidelines

Buy-in by providers at other levels of care who are in supportive roles (e.g. nursing, pharmacy, laboratory) and hospital administrators	Task force members met with hospital leaders to explain rationale for intervention and demonstrate leadership endorsement

Designing a practical and effective protocol suited to institutional needs and capabilities	Task force conducted a multidisciplinary assessment of hospital expertise and practice pattern including nursing and anesthesiologist skill in the use of intravenous insulin. Protocols were developed and piloted and further refined prior to institution-wide adoption

Obtaining resources required for program success, including point-of-care glucose meters in preoperative, intraoperative, and postoperative care areas	Representative of the task force worked directly with hospital administrators for funding required for infrastructure and equipment

Staff training, including nursing, anesthesia, and endocrine staff regarding the protocol as well as specific skills in infusion therapy and glucose meters	Nurse educators, pharmacists, and endocrinologists performed education for support staff in the perioperative area. Provider education by each department and leadership group

Ensuring uniformity and ease of daily protocol use	Consistent elements were put in place: computerized order sets, written protocols available in the perioperative areas and 24/7 pager access to a designated physician for support

Protocol maintenance and improvement	Scheduled reviews of efficacy and safety with members from representative departments, easily identifiable point person who can be contacted with questions, concerns, and suggestions

**Table 3:** Comparisons between Boston Medical Center and Yale New Haven Protocols.

	Boston medical center	Yale new haven
Protocol leadership	Endocrinology, anesthesiology, nursing, pharmacy, and surgery	Endocrinology, intensivist, anesthesiology, nursing, pharmacy, surgery, and administrators
Target intraoperative glucose range	120–180 mg/dL	120–180 mg/dL
Threshold for treatment of perioperative hyperglycemia	>180 mg/dL	>200 mg/dL (pre-op) >180 mg/dl (intra- and post-op)
Threshold for evaluation of metabolic stability preoperatively	>300 mg/dL	At the discretion of the practitioner
Recommendation for cancellation of nonurgent surgery*	>500 mg/dL	>400 mg/dL

*See text for details. Surgery could also be cancelled at the discretion of the provider at a different glucose level based on surgical urgency and procedure risk.

## References

[1] Smiley D, Umpierrez GE (2010). Management of hyperglycemia in hospitalized patients. *Annals of the New York Academy of Sciences*.

[2] Vriesendorp TM, Morélis QJ, DeVries JH, Legemate DA, Hoekstra JBL (2004). Early post-operative glucose levels are an independent risk factor for infection after peripheral vascular surgery. A retrospective study. *European Journal of Vascular and Endovascular Surgery*.

[3] Pomposelli JJ, Baxter JK, Babineau TJ (1998). Early postoperative glucose control predicts nosocomial infection rate in diabetic patients. *Journal of Parenteral and Enteral Nutrition*.

[4] Swenne CL, Lindholm C, Borowiec J, Schnell AE, Carlsson M (2005). Peri-operative glucose control and development of surgical wound infections in patients undergoing coronary artery bypass graft. *Journal of Hospital Infection*.

[5] Noordzij PG, Boersma E, Schereiner F (2007). Increased preoperative glucose levels are associated with perioperative mortality in patients undergoing noncardiac, nonvascular surgery. *European Journal of Endocrinology*.

[6] Schmeltz LR, DeSantis AJ, Thiyagarajan V (2007). Reduction of surgical mortality and morbidity in diabetic patients undergoing cardiac surgery with a combined intravenous and subcutaneous insulin glucose management strategy. *Diabetes Care*.

[7] Akhtar S, Barash PG, Inzucchi SE (2010). Scientific principles and clinical implications of perioperative glucose regulation and control. *Anesthesia & Analgesia*.

[8] Gandhi GY, Nuttall GA, Abel MD (2005). Intraoperative hyperglycemia and perioperative outcomes in cardiac surgery patients. *Mayo Clinic Proceedings*.

[9] Frisch A, Chandra P, Smiley D (2010). Prevalence and clinical outcome of hyperglycemia in the perioperative period in noncardiac surgery. *Diabetes Care*.

[10] Polito A, Thiagarajan RR, Laussen PC (2008). Association between intraoperative and early postoperative glucose levels and adverse outcomes after complex congenital heart surgery. *Circulation*.

[11] King JT, Goulet JL, Perkal MF, Rosenthal RA (2010). Glycemic control and infections in patients with diabetes undergoing noncardiac surgery. *Annals of Surgery*.

[12] Subramaniam B, Panzica PJ, Novack V (2009). Continuous perioperative insulin infusion decreases major cardiovascular events in patients undergoing vascular surgery: a prospective, randomized trial. *Anesthesiology*.

[13] Umpierrez GE, Smiley D, Jacobs S (2011). Randomized study of basal-bolus insulin therapy in the inpatient management of patients with type 2 diabetes undergoing general surgery (RABBIT 2 surgery). *Diabetes Care*.

[14] Gandhi GY, Nuttall GA, Abel MD (2007). Intensive intraoperative insulin therapy versus conventional glucose management during cardiac surgery: a randomized trial. *Annals of Internal Medicine*.

[15] Umpierrez GE, Hor T, Smiley D (2009). Comparison of inpatient insulin regimens with detemir plus aspart Versus neutral protamine hagedorn plus regular in medical patients with type 2 diabetes. *Journal of Clinical Endocrinology and Metabolism*.

[16] Umpierrez GE, Smiley D, Zisman A (2007). Randomized study of basal-bolus insulin therapy in the inpatient management of patients with type 2 diabetes (RABBIT 2 Trial). *Diabetes Care*.

[17] Lleva RR, Inzucchi SE (2011). Hospital management of hyperglycemia. *Current Opinion in Endocrinology, Diabetes and Obesity*.

[18] Hovorka R, Chassin LJ, Ellmerer M, Plank J, Wilinska ME (2008). A simulation model of glucose regulation in the critically ill. *Physiological Measurement*.

[19] Friedewald WT, Buse JB, Bigger JT (2008). Effects of intensive glucose lowering in type 2 diabetes. *The New England Journal of Medicine*.

[20] Rea RS, Donihi AC, Bobeck M (2007). Implementing an intravenous insulin infusion protocol in the intensive care unit. *American Journal of Health-System Pharmacy*.

[21] Soylemez Wiener R, Wiener DC, Larson RJ (2008). Benefits and risks of tight glucose control in critically ill adults: a meta-analysis. *Journal of the American Medical Association*.

[22] Wilson M, Weinreb J, Soo Hoo GW (2007). Intensive insulin therapy in critical care: a review of 12 protocols. *Diabetes Care*.

[23] Ku SY, Sayre CA, Hirsch IB, Kelly JL (2005). New insulin infusion protocol Improves blood glucose control in hospitalized patients without increasing hypoglycemia. *Joint Commission Journal on Quality and Patient Safety*.

[24] Blaha J, Kopecky P, Matias M (2009). Comparison of three protocols for tight glycemic control in cardiac surgery patients. *Diabetes Care*.

[25] Krinsley JS (2004). Effect of an intensive glucose management protocol on the mortality of critically Ill adult patients. *Mayo Clinic Proceedings*.

[26] DeSantis AJ, Schmeltz LR, Schmidt K (2006). Inpatient management of hyperglycemia: the northwestern experience. *Endocrine Practice*.

[27] Krikorian A, Ismail-Beigi F, Moghissi ES (2010). Comparisons of different insulin infusion protocols: a review of recent literature. *Current Opinion in Clinical Nutrition and Metabolic Care*.

[28] Inzucchi SE (2006). Management of hyperglycemia in the hospital setting. *The New England Journal of Medicine*.

[29] Korytkowski M, Moghissi ES (2009). Treatment options for safely achieving glycemic targets in the hospital. *Revisiting Inpatient Hyperglycemia New Recommendations, Evolving Data, and Practical Implications for Implementation*.

[30] Finfer S, Bellomi R, Blair D (2009). Intensive versus conventional glucose control in critically Ill patients. *The New England Journal of Medicine*.

[31] Griesdale DEG, de Souza RJ, van Dam RM (2009). Intensive insulin therapy and mortality among critically ill patients: a meta-analysis including NICE-SUGAR study data. *Canadian Medical Association Journal*.

[32] Marik PE, Preiser JC (2010). Toward understanding tight glycemic control in the ICU: a systematic review and metaanalysis. *Chest*.

[33] Kansagara D, Fu R, Freeman M, Wolf F, Helfand M (2011). Intensive insulin therapy in hospitalized patients: a systematic review. *Annals of Internal Medicine*.

[34] Moghissi ES, Korytkowski MT, DiNardo M (2009). American Association of Clinical Endocrinologists and American Diabetes Association consensus statement on inpatient glycemic control. *Diabetes Care*.

[35] Poldermans D, Bax JJ, Boersma E (2009). Guidelines for pre-operative cardiac risk assessment and perioperative cardiac management in non-cardiac surgery. *European Heart Journal*.

[36] Dellinger RP, Levy MM, Carlet JM (2008). Surviving sepsis campaign: international guidelines for management of severe sepsis and septic shock: 2008. *Critical Care Medicine*.

[37] Kushner FG, Hand M, Smith SC (2009). 2009 focused updates: ACC/AHA guidelines for the management of patients with st-elevation myocardial infarction (Updating the 2004 guideline and 2007 focused update) and ACC/AHA/SCAI guidelines on percutaneous coronary intervention (Updating the 2005 Guideline and 2007 Focused Update)): a report of the American College of Cardiology Foundation/American Heart Association Task Force on Practice Guidelines. *Circulation*.

[38] Kavanagh BP, McCowen KC (2010). Glycemic control in the ICU. *The New England Journal of Medicine*.

[39] Qaseem A, Humphrey LL, Chou R, Snow V, Shekelle P (2011). Use of intensive insulin therapy for the management of glycemic control in hospitalized patients: a clinical practice guideline from the American College of Physicians. *Annals of Internal Medicine*.

[40] Svensson AM, McGuire DK, Abrahamsson P, Dellborg M (2005). Association between hyper- and hypoglycaemia and 2 year all-cause mortality risk in diabetic patients with acute coronary events. *European Heart Journal*.

[41] Pinto DS, Skolnick AH, Kirtane AJ (2005). U-shaped relationship of blood glucose with adverse outcomes among patients with ST-segment elevation myocardial infarction [1]. *Journal of the American College of Cardiology*.

[42] Egi M, Bellomo R, Stachowski E (2010). Hypoglycemia and outcome in critically ill patients. *Mayo Clinic Proceedings*.

[43] Kosiborod M, Inzucchi SE, Goyal A (2009). Relationship between spontaneous and iatrogenic hypoglycemia and mortality in patients hospitalized with acute myocardial infarction. *Journal of the American Medical Association*.

[44] Siegelaar SE, Hermanides J, Oudemans-van Straaten HM (2010). Mean glucose during ICU admission is related to mortality by a U-shaped curve in surgical and medical patients: a retrospective cohort study. *Critical Care*.

[45] Vriesendorp TM, DeVries JH, Van Santen S (2006). Evaluation of short-term consequences of hypoglycemia in an intensive care unit. *Critical Care Medicine*.

[46] Dungan K, Chapman J, Braithwaite SS, Buse J (2007). Glucose measurement: confounding issues in setting targets for inpatient management. *Diabetes Care*.

[47] Kanji S, Buffie J, Hutton B (2005). Reliability of point-of-care testing for glucose measurement in critically ill adults. *Critical Care Medicine*.

[48] Rice MJ, Pitkin AD, Coursin DB (2010). Glucose measurement in the operating room: more complicated than it seems. *Anesthesia & Analgesia*.

[49] Peterfreund RA, Akhtar S (2010). Editorial: how sweet it is ... (or isn't)!. *Anesthesia & Analgesia*.

[50] Pietras SM Glycemic control in the peri-operative setting: results of a 3 month pilot program at an academic institution.

